# The effect of North Carolina free clinics on hospitalizations for ambulatory care sensitive conditions among the uninsured

**DOI:** 10.1186/s12913-018-3082-1

**Published:** 2018-04-12

**Authors:** Jenny Hutchison, Michael E. Thompson, Jennifer Troyer, Christine Elnitsky, Maren J. Coffman, M. Lori Thomas

**Affiliations:** 1 School of Social Work, University of North Carolina at Charlotte, 9201 University City Blvd, Charlotte, NC 28223-0001 USA; 2 Public Health Sciences, University of North Carolina at Charlotte, 9201 University City Blvd, Charlotte, NC 28223-0001 USA; 3 Department of Economics, Belk College of Business, University of North Carolina at Charlotte, 9201 University City Blvd, Charlotte, NC 28223-0001 USA; 4 School of Nursing, University of North Carolina at Charlotte, 9201 University City Blvd, Charlotte, NC 28223-0001 USA; 5 School of Social Work, University of North Carolina at Charlotte, 9201 University City Blvd, Charlotte, NC 28223-0001 USA

**Keywords:** Uninsured adults, Primary care, Ambulatory care sensitive conditions, Free clinics

## Abstract

**Background:**

Free clinics are volunteer based organizations that provide health care services to low-income individuals for free or minimal cost. Communities served by a free clinic can provide ambulatory care services for uninsured individuals, reducing reliance on costly hospital admissions for ambulatory care sensitive conditions. This study examines whether free clinics in North Carolina reduce hospitalizations for ambulatory care sensitive conditions for uninsured adults.

**Methods:**

The study used North Carolina hospital discharge data from 2003 to 2007, restricted to uninsured adults residing in North Carolina (*N* = 270,325). Prevention Quality Indicators identified hospitalizations for ambulatory care sensitive conditions. The entry of new free clinics in some counties during this time period in conjunction with county-level and year fixed effects allows the logistic regression analysis to simulate a pre/post study design.

**Results:**

Discharges for ambulatory care sensitive conditions constituted 12.6% of the sample. Despite the limited coverage provided by free clinics, which serve 5.5% of the uninsured in North Carolina, uninsured adults in counties served by a free clinic had an 8.0% reduced odds of a hospitalization being for an ambulatory care sensitive condition. When the model is limited to ambulatory care sensitive conditions related to chronic conditions, the odds of a hospitalization of an uninsured adult for an ambulatory care sensitive condition in counties served by a free clinic is reduced by 9.0%.

**Conclusion:**

Free clinics are effective providers of primary care services for uninsured individuals, particularly for those with chronic conditions. To enhance this impact by increasing free clinics’ reach, state and local policy makers should support and encourage development of free clinics in high need areas.

**Electronic supplementary material:**

The online version of this article (10.1186/s12913-018-3082-1) contains supplementary material, which is available to authorized users.

## Background

Conditions that are treated in an acute care setting that could have been mitigated through access to appropriate primary care are known as Ambulatory Care Sensitive (ACS) conditions. Lack of access to primary care results in the absence of regular preventive care, monitoring of chronic illnesses, and early treatment of acute conditions [1, 2]. As untreated health conditions worsen, hospitalization may be required; examples include uncontrolled asthma triggering a lung infection or unmanaged diabetes resulting in a stroke. High rates of ACS conditions that result in admission to hospitals or emergency departments are therefore an indicator of poor access to primary care.

Despite modest improvements in the number of preventable hospitalizations for ACS conditions in recent years, total costs for potentially preventable hospitalizations are estimated to exceed $30 billion annually [3]. Uninsured adults, who are less likely to have regular source of care and more likely to have unmet medical needs [4], are hospitalized for ACS conditions more frequently than Medicaid recipients or individuals with commercial insurance [5]. Efforts to improve primary care access for populations with the highest rates of hospitalizations for ACS conditions, such as the uninsured, could aid in reducing aggregate health care costs in the U.S. [6].

Free clinics, which provide medical care for free or minimal cost, are one avenue to address the medical needs of the uninsured. While free clinics do not have the resources to meet all the medical needs of these populations, they often provide care for chronic illnesses through regular monitoring, dispensing medications, and providing lab tests. Their services may be more limited than those of other primary care organizations that accept insurance due to limited funding for free clinics and reliance on medical and administrative volunteers. Clients served by free clinics frequently come from demographic groups identified as having an increased likelihood of being admitted for ACS conditions, such as uninsured [5], individuals from low-income areas [6, 7], and minorities [8, 9].

This study examines whether free clinics in North Carolina (NC) reduce hospitalizations for ACS conditions for uninsured adults. More precisely, this work considers whether hospitalization of an uninsured adult in a community served by a free clinic has lower odds of being admitted for an ACS condition. We also consider whether this relationship is stronger for ACS hospitalizations resulting from chronic conditions as compared to ACS hospitalizations resulting from acute conditions.

### Literature review

As the Affordable Care Act (ACA) continues to unfold, the US healthcare system is focused on serving the medical needs of the newly insured through the exchanges and expanded Medicaid enrollment. Reduced funding under the ACA for uncompensated care at safety net facilities, such as public hospitals [10, 11], and increased demand in primary care offices, particularly from lower paying Medicaid beneficiaries [12], could result in fewer safety net options for those who remain uninsured, and, therefore, create a greater reliance on free clinics for these vulnerable groups. Furthermore, Republican control of congress and the presidency following the 2016 elections make long promised revisions to, if not outright repeal of, the ACA inevitable. Such changes could result in higher rates of uninsured adults, especially among the working poor and near poor [13].

Several studies describe free clinics and their patients [14–17]. Not surprising, the populations served are between the ages of 18 and 64, female, uninsured, and living below the Federal Poverty Level (FPL) [14, 16–18]. Free clinics lack resources, organizational structure, and services compared to their mainstream counterparts, but have an important and enduring role within the US healthcare delivery system [14, 15, 17, 18].

Most studies examining health outcomes or access to care in relation to free clinics typically relied on a single clinic or small cluster of clinics. Specifically studies found free clinic patients realized improvement in chronic disease management such as increased exercise [19], reduced HgbA1c, lower LDL levels, and lower blood pressure [20], high rates of recommended care (96% received HbA1c monitoring and 80% received nephropathy monitoring), and blood pressure control [21]. Although these pilot studies, occurring in a single clinic with a small sample, have limited generalizability, they offer support for free clinics’ ability to contribute to improved health outcomes for populations that are likely to be at higher risk of chronic illnesses.

One large-scale study assessed the impact of free clinics on low income or elderly hospitalized patients in Virginia (including uninsured, Medicaid, and Medicare recipients) [22]. Communities with a free clinic had lower rates of preventable hospitalizations than communities without a free clinic, but the association was only marginally significant [22]. Given that free clinics generally do not serve individuals with third party or public insurance, the inclusion of uninsured discharges with those funded by public insurance does not allow for a direct examination of the impact of free clinics on the uninsured. In addition, the measure of hospitalization for an ACS condition utilized in the Virginia study predates the Agency for Healthcare Research and Quality (AHRQ) indices for ACS conditions known as the Prevention Quality Indicators (PQI) [23]. AHRQ assembled a workgroup to assess individual and composite indicators in terms of the existing literature, validity and precision, which expanded upon the earlier studies used as the basis for determining ACS conditions in the above referenced study [23, 24]. Our study is the first large-scale, multi-year examination of the impact of free clinics on hospitalization for ACS conditions for the uninsured population using PQIs to measure ACS conditions.

## Methods

Cecil P. Sheps Center for Health Services Research provided NC hospital discharge data for 2003 through 2007. From 2003 to 2007, eighteen free clinics opened in NC [25] (Additional file 1). The sample was restricted to NC adults who were designated self-pay (*n* = 270,325).

### Dependent variables

The dependent variable was whether a given hospitalization was for an ACS condition that was preventable with adequate primary care. Preventable ACS conditions were identified using the AHRQ’s overall PQI#90, based on principal diagnosis codes.

ACS conditions were further divided into two separate indicators: ACS conditions related to chronic illness (PQI#92) and ACS conditions related to acute conditions (PQI#91). Chronic illnesses require ongoing regular medical care while ACS conditions related to acute conditions require time sensitive treatment to avoid hospitalization. Table 1 below lists the conditions included for PQI #91 and #92.

**Table 1:** Chronic and acute conditions included in AHRQ Prevention Quality Indicators*

ACS conditions related to chronic illness (PQI #92)	
diabetes with short-term complications	Diabetes with long-term complications
uncontrolled diabetes without complications	Diabetes with lower-extremity amputation
chronic obstructive pulmonary disease	Asthma
hypertension	Heart failure
angina without a cardiac procedure	
ACS conditions related to acute conditions (PQI#91)	
dehydration	Bacterial pneumonia
urinary tract infections	

*AHRQ’s overall PQI includes all 12 conditions listed above

Source: Agency for Healthcare Research and Quality. Quality indicator User Guide: Preventions quality indicators (PQI) composite measures Rockville, MD: US Department of Health & Human Services; 2011

### Independent variables

The key independent variable was a dichotomous indicator of whether a discharged patient resided in a county served by a free clinic that had been in operation for at least a year. We anticipated that free clinics would reduce the odds that an uninsured patient is hospitalized for an ACS condition. In addition, given the focus of free clinics, we expected the effect to be meaningful for chronic ACS conditions and negligible for acute ACS conditions. Clinic service areas correspond to the clinic’s response to a question on the annual North Carolina Association of Free Clinics (NCAFC) outcome survey asking which counties are included in their service areas. Free clinics are defined as health care safety net organizations that are a 501(c) [3] tax-exempt organization or an affiliate of such an organization, provide medical care for free or minimal donation, and do not accept third party insurance. The indicator was limited to those clinics that provided medical services. Table 2 lists the number of medical free clinics operating in each year and number of counties served by a free clinic. North Carolina consists of one hundred counties.

**Table 2:** Free clinics operating for at least one year and number of counties served: 2003–2007

Year	Free clinics operating for at least 1 year	Previously un-served counties *	Total NC counties served by a free clinic
2003	46	N/A	50
2004	47	6	56
2005	55	4	60
2006	59	7	67
2007	64	6	73
Total	N/A	23	N/A

*Only medical free clinics are included. A free clinic opening may result in the addition of more than one county being served by a clinic if the clinic serves neighboring counties

Covariates included in the model were based on conceptualization of health services utilization, controlling for the co-existing influences of individual, community and health services environment on an individual’s healthcare utilization [26]. Individual demographic variables, including age, gender, and race, were used. While race is typically collected as part of hospital administrative data, it was not a required element for NC hospitals until 2010. Consequently, nearly 40% of observations in the sample lack race information. Observations without race data were retained and categorized separately.

Community level factors included percentage uninsured, percentage minority composition, and percentage living below the federal poverty level. The health service environment was represented in the model by the number of hospital beds per 1000 population and the number of MDs per 10,000 population for each county. A dichotomous variable indicating the presence of a Federally Qualified Health Center (FQHC) in a county represented other available safety net facilities, where FQHCs serve low-income populations with and without insurance using a sliding fee scale. Both community and health service environment factors were extracted from the Area Health Resource files. Table 3 below lists each covariate included in the model, the definition and the data source the information was extracted from.

**Table 3:** Data sources for all variables

Variable	Definition	Source
Counties Served by a Free Clinic	A county with at least one free clinic in operation for at least one year.	North Carolina Association of Free Clinics databases and annual clinic survey
ACS condition	Identified using AHRQ’s PQI. PQI #9 0 – all ACS conditions; PQI # 91 – ACS Acute conditions; PQI # 92 – ACS chronic conditions	North Carolina Hospital Discharge Data
Sex	Male or female	North Carolina Hospital Discharge Data
Age	Categorized into 5 groups: 18 to 29, 30 to 39, 40 to 49, 50 to 64, & 65 or older	North Carolina Hospital Discharge Data
Race	White, Black, Asian, Native American, or other	North Carolina Hospital Discharge Data
FQHC	1 or more FQHCs in the county	Area Health Resource File
Hospital Beds per 1000 population	Acute care hospital beds per 1000 population	Area Health Resource File
MDs per 10,000 population	# of MDs per 10,000 population	Area Health Resource File
Percent minority	Proportion of the population non-white.	Area Health Resource File
Percent no health insurance	% of the population under 65 w/o health insurance	Area Health Resource File
Percent living in poverty	% of the population living below the poverty level	Area Health Resource File

### Analysis

The use of a multi-year dataset and the entry of free clinics in some counties over time, allowed for logistic regression analysis with county-level and year fixed effects to be analogous to a difference-in-differences approach. Analysis using ordinary least squares with fixed county and year effects also was conducted; those qualitatively similar results are not reported. By controlling for each time period and each county, the binary indicator for the presence of a free clinic in a particular county in a particular year may be interpreted as the causal effect of having a free clinic in the county on the odds that a hospitalization is for an ACS condition. Analyses were conducted for all three ACS condition outcomes.

## Results

Descriptive statistics for the hospitalizations as well as demographic and community characteristics for the uninsured sample are included in Table 4. Discharges for ACS conditions constituted 12.6% of the sample, with approximately two-thirds of the ACS conditions attributable to chronic illnesses (not shown). Most uninsured hospitalizations were in counties that were served by a free clinic. Hospitalization for men slightly exceeded those for women (52.7% men vs. 47.3% women). Although those between ages 40 and 49 (26.9%) had the highest proportion of discharges, the sizes of the groups under age 65 were similar. For hospitals reporting race, White American comprised the largest group (33.6%).

**Table 4:** Demographic and community characteristics of total sample and by discharge type

	Total (270,325)	ACS Condition (34,195)	Non-ACS Condition (236,130)	
	%	%	%	*P*-value*
County served by a Free Clinic	76.9	75.8	77.0	Reference
No Free Clinic	23.1	24.2	23.0	< 0.001
Sex				
Female	47.3	47.5	47.2	Reference
Male	52.7	52.5	52.8	0.325
Age				
18 to 29	24.7	16.3	26.0	Reference
30 to 39	21.8	17.7	22.4	< 0.001
40 to 49	26.9	30.1	26.5	< 0.001
50 to 64	25.0	33.5	23.7	< 0.001
> 65	1.5	2.4	1.4	< 0.001
Race				
White	33.6	27.7	34.5	Reference
Black	17.6	23.6	16.7	< 0.001
Asian	0.8	0.3	0.9	< 0.001
Native American	1.3	1.1	1.4	0.788
Other	7.9	4.0	8.5	< 0.001
Missing	38.7	43.3	38.0	< 0.001
FQHC(s) in County	52.2	49.8	52.5	Reference
No FQHCs	47.9	50.2	47.5	< 0.001
	Mean	Mean	Mean	
Beds per 1000 pop	3.4	3.5	3.4	< 0.001
MDs per 10,000 pop	2.6	2.6	2.6	< 0.001
Percent Minority	33.2	33.8	33.1	< 0.001
Percent Living in Poverty	14.8	15.1	14.8	< 0.001
Percent w/o Insurance	17.6	17.5	17.7	< 0.001

*The *p*-values are for tests for differences between hospitalizations with and without an ACS condition in the proportion or mean

Table 4 also presents the demographic and community characteristics of the sample by whether the hospitalization was for an ACS condition or not. Hospitalizations for ACS conditions occurred more frequently in counties with a free clinic and without an FQHC. Uninsured men and women were hospitalized with an ACS condition at the same rate. However, uninsured middle-aged, older and Black Americans were more often hospitalized with an ACS condition as compared to their reference group (i.e., younger adults and White Americans, respectively). Although t-tests indicated the number of hospital beds, number of MDs, percent of the population minority, percent of the population living in poverty and the percent of the population without health insurance differed statistically for the two groups, actual differences were minimal in practical terms.

The results from the full models, which controlled for fixed effects across years and counties, for the three outcomes – hospitalization for an ACS condition, hospitalization for an ACS condition related to chronic illness, and hospitalization for an ACS condition related to an acute condition - are presented in Table 5. The model supports the hypothesis that free clinics aid in decreasing the odds of a hospitalization for an ACS condition for uninsured individuals in the communities they serve; a hospitalization for an uninsured individual residing in a county served by a free clinic had an 8.0% reduced odds of being for an ACS condition. Furthermore, the model confirms the secondary hypothesis that free clinic services are more effective in aiding the uninsured with chronic illness management, versus acute illneses, as evidenced by the 9.0% reduction in the odds of hospitalization for a chronic ACS condition.

**Table 5:** Odds ratios for the likelihood that an uninsured hospitalization was for an ACS condition

	Chronic or Acute ACS condition	Chronic ACS condition	Acute ACS condition
	(*N* = 270,325)	(*N* = 270,325)	(*N* = 270,200)*
	AOR (95% CI)	AOR (95% CI)	AOR (95% CI)
County served by Free Clinic(s)	0.92 (0.86,0.99)	0.91 (0.84,0.99)	0.97 (0.86,1.09)
Female	1.08 (1.05,1.10)	0.91 (0.89,0.94)	1.48 (1.42,1.54)
Age			
30 to 39	1.23 (1.18,1.28)	1.30 (1.24,1.37)	1.09 (1.03,1.16)
40 to 49	1.70 (1.64,1.77)	1.91 (1.83,2.00)	1.26 (1.19,1.33)
50 to 64	2.12 (2.05,2.20)	2.47 (2.37,2.58)	1.40 (1.32,1.48)
> 64	2.90 (2.67,3.15)	2.95 (2.67,3.26)	2.31 (2.03,2.63)
Race			
Black	1.84 (1.78,1.91)	2.15 (2.07,2.24)	1.14 (1.07,1.21)
Asian	0.62 (0.51,0.74)	0.64 (0.51,0.81)	0.60 (0.44,0.81)
Native American	1.10 (0.97,1.24)	1.06 (0.91,1.23)	1.16 (0.95,1.41)
Other	0.74 (0.69,0.78)	0.74 (0.69,0.80)	0.73 (0.66,0.80)
Missing	1.55 (1.50,1.60)	1.60 (1.54,1.67)	1.35 (1.28,1.43)
FQHC(s) in County	1.06 (0.96,1.17)	1.06 (0.94,1.19)	1.06 (0.89,1.25)
Hospital beds per 1000 pop	0.97 (0.91,1.03)	0.95 (0.88,1.02)	1.00 (0.91,1.11)
MDs per 10,000 pop	0.99 (0.92,1.03)	0.95 (0.89,1.02)	1.03 (0.94,1.13)
% living in Poverty	1.00 (0.99,1.01)	1.00 (0.99,1.02)	0.99 (0.97,1.01)
% w/o health Insurance	0.98 (0.97,1.00)	0.97 (0.95,0.98)	1.03 (1.00,1.06)
% Minority	3.51(0.21,57.89)	4.21(0.16,111.88)	1.57(0.01,195.62)

*AOR:* Adjusted Odds Ratio

*Analysis for acute ACS conditions does not include one county, which had no hospitalizations for acute ACS conditions. The sample for the acute analysis is 270,200

The lack of significance of the odds ratio for free clinics when examining hospitalization related to an acute ACS condition is consistent with the notion that free clinics are more focused on providing preventive care for chronic conditions than addressing time sensitive acute conditions. None of the community factors were statistically significant, likely due to the lack of variation in these measures within counties over time in the period examined.

Examining all ACS conditions, uninsured women had slightly higher odds of being hospitalized for an ACS condition as compared to uninsured men (OR women: 1.08). Increasing age was associated with steadily increasing odds of an ACS hospitalization for the uninsured (OR 30 to 39: 1.23; OR 40 to 49: 1.67; OR 50 to 64: 2.12; and OR 65 or older: 2.90). In addition, uninsured Black Americans had 1.84 times the odds of being admitted for an ACS condition as compared to uninsured White Americans, while Asian Americans had a 38% reduced odds of being admitted for an ACS condition versus White Americans.

For ACS conditions related to chronic illnesses, the magnitude of the odds ratios for uninsured middle-aged, older, and Black Americans increased relative to the odds ratios for all ACS conditions. Black Americans experience over twice the odds (OR: 2.15) of being hospitalized for ACS conditions related to chronic illnesses compared to White Americans. The odds ratios for the age categories for chronic illness related ACS conditions followed a similar pattern as those for overall ACS conditions, but the effect was greater for each age category. However, in limiting ACS conditions to only those related to chronic illnesses, women had 9.0% lower odds of being hospitalized for a chronic condition versus their male counterparts.

## Discussion

Uninsured individuals with limited access to primary care are at greater risk of being hospitalized for ACS conditions [5, 27], incurring potentially unnecessary costs for hospitals and health care systems. However, few large-scale, multi-year studies have focused on how free clinics affect hospitalizations for ACS conditions for uninsured adults. This study finds that proximity to a free clinic significantly reduces an uninsured individual’s odds of a hospitalization for an ACS condition.

This large-scale study is the first to examine the impact of free clinics serving a community on hospitalizations for uninsured individuals with ACS conditions during a period when multiple new clinics opened. The incorporation of the county-level and year fixed effects creates a pre/post study design, with the results driven by counties gaining free clinic services during the study period. The study design allows us to argue that our finding is causal in nature. During the time period examined (2003 to 2007), 18 new free clinics were opened in NC serving an additional 23 counties. Although free clinics only serve approximately 87,000 uninsured [28], equating to approximately 5.5% of the uninsured adults in NC (pre-ACA) [29], the model indicates they contribute to a statistically and practically significant reduction in the odds of a hospitalization for an ACS condition by an uninsured individual, an effect which would be increasingly magnified as the proportion of uninsured served increases.

We found that FQHCs had no significant effect on the odds of hospitalization for an ACS condition for the uninsured. This finding is consistent with other research on the uninsured [30, 31], implying that FQHCs may be more successful at providing primary care to Medicaid and/or Medicare recipients than to uninsured individuals. Administrative and/or economic requirements for care at FQHCs could impede access for uninsured individuals [32, 33]. However, the result in our study may be due to limited entry of FQHCs over the period examined. To the extent that FQHCs are not providing accessible or adequate primary care for the uninsured, other providers, such as free clinics, are necessary to minimize avoidable and costly use of hospitals for ACS conditions.

As expected, free clinics’ contribution to providing medical care for the uninsured appears to be most pronounced in aiding management of chronic diseases. Patients at free clinics may benefit from ongoing regular contact with a provider enabling them to diagnose chronic conditions, maintain prescriptions, adjust treatment as needed, and recognize symptoms of declining health. For acute ACS conditions, limited appointment availability and a lack of specialists and equipment may restrict free clinics’ ability to address time sensitive care needs.

While care for acute conditions is a necessary part of primary care treatment, the provision of adequate ongoing regular medical care for chronic illness is an important need for low income populations, who are at higher risk of having one or more chronic illnesses. Previous studies of individual free clinics have shown improved self-care management among the uninsured in the form of increased exercise time, improved blood pressure control, and reducing HgbA1c levels [19–21], supporting free clinics’ focus on addressing chronic illnesses amongst the uninsured. Further investigation of free clinics’ programming may uncover practices that could be adopted at other health care safety net organizations in treating chronic illnesses for the uninsured.

The current study highlights the interconnectedness of chronic illness among the uninsured and hospitalizations for ACS conditions. Our study found that uninsured Black Americans had higher odds of being hospitalized for ACS conditions, with the effect larger for chronic ACS conditions. This pattern is consistent with prior research indicating increased rates of hospitalization for chronic illness related ACS conditions for Black Americans versus rates of hospitalizations for all or only acute ACS conditions [9]. We also found that middle-aged and older uninsured individuals had higher odds of being hospitalized for ACS conditions, with the effect larger for chronic ACS conditions. Hospitalizations for ACS conditions for middle-aged adults create added costs from lost days of work, and higher out-of-pocket costs that can have long-term ramifications for the individual and their families [8]. These demographic groups (Black, middle-aged and older Americans) are at greater risk for having one or more chronic illnesses [34–36], and with limited access to health care as a result of lack of insurance, they are likely to have unattended conditions that require costly hospitalizations. Future research is warranted to understand whether disease management programs at free clinics are effective in improving outcomes for uninsured Black Americans, middle-aged, and older adults with one or more chronic illnesses.

Finally, the current study again confirms the ongoing inequities in health and health care for Black Americans. Uninsured Black Americans had an 84.0% increased odds of being hospitalized for an ACS condition as compared to White Americans. This finding is consistent with earlier studies finding of higher rates of ACS hospitalizations for Black Americans for all types of payers [8, 9, 37]. Organizations within the health care safety net need to develop partnerships with social organizations directed toward, operated by, and frequented by Black Americans to improve access as well as improve our understanding of barriers to care for Black Americans.

### Limitations

This study has several limitations. First, the study has no information concerning where individuals sought primary care or what percentage of care for a given county was provided by a free clinic. Second, the covariates for the health service environment are at the county level. However, county boundaries are not equivalent to service areas. Individuals in a specified county may have access to hospitals, FQHCs, or physicians in a neighboring county. If FQHCs served uninsured individuals outside of their immediate county, it is uncertain how it would affect the odds ratio for free clinics, given FQHCs could be serving uninsured in counties served by the free clinics and/or counties not served by the free clinics. Finally, the study utilized data from NC, and may not be generalizable to other states.

## Conclusion

Although uninsured hospital stays for ambulatory care sensitive conditions are twice as common as ACS hospital stays for Medicaid or private insurance [5], few studies have examined whether free clinics aid in reducing the odds that an uninsured individual is hospitalized for an ACS condition by providing effective primary care. Despite the success in reducing the number of uninsured in the US with the implementation of the ACA, states that rejected the Medicaid expansion will continue to have higher rates of uninsured, and therefore potentially higher rates of ACS admissions. This study indicates that free clinics dedicated to providing care for uninsured adults in NC, despite serving less than 6% of the uninsured, contribute to statistically and practically significant lower proportion of hospitalizations for ACS conditions by the uninsured.

Free clinics in NC have been successful in addressing the needs of uninsured adults with chronic conditions. However, given the increased odds of being hospitalized for an ACS condition related to a chronic illness for middle-aged, older and Black Americans, further research is warranted on the effectiveness of free clinics in meeting the needs of these groups.

As NC and other southern states continue to opt out of the Medicaid expansion, and in the absence of universal health care, these states need to investigate how to make primary care accessible to the uninsured in order to improve their health and minimize costly hospital use for ACS conditions. To increase free clinics’ reach, state and local policy makers should encourage development of free clinics in high need areas, such as low income and minority communities. Meeting the health care needs of the uninsured could improve health outcomes for the uninsured while reducing healthcare costs for the community.

## Additional file

Additional file 1: Confirmation of free clinics. Processes used to identify free clinics. (DOCX 12 kb)

## Abbreviations

ACA: Affordable Care Act
ACS: Ambulatory Care Sensitive
AHRQ: Agency for Healthcare Research and Quality
AOR: Adjusted Odds Ratio
FPL: Federal Poverty Level
FQHC: Federally Qualified Health Center
NC: North Carolina
NCAFC: North Carolina Association of Free Clinics
PQI: Prevention Quality Indicators
