# Supplementary material for: The effect of North Carolina free clinics on hospitalizations for ambulatory care sensitive conditions among the uninsured
Source: BMC Health Serv Res. 2018 Apr 12;18:280. doi: 10.1186/s12913-018-3082-1 (PMC5897934; doi:10.1186/s12913-018-3082-1)
Supplement: Supplementary file 1 — Confirmation of free clinics. Processes used to identify free clinics. (DOCX 12 kb) [file 12913_2018_3082_MOESM1_ESM.docx]

**Additional File 1**

Title of Data: Confirmation of free clinics

Description of Data: Processes used to identify free clinics

During the period 2003 to 2007, 83 clinics were members of North Carolina Association of Free Clinics (NCAFC). Base grants offered to existing clinics through the partnership with Blue Cross Blue Shield Foundation are a major incentive for free clinics to become members of the NCAFC and encourage the vast majority of North Carolina’s free clinics to join. The Association provided addresses and opening year information for current and past member clinics. Historical files at the NCAFC were reviewed to insure clinics that were operating during the 2003-2007 period, but subsequently closed were included. Several methods were used to confirm the historical locations and opening year of a clinic including reviewing NCAFC files, searching the North Carolina Department of Secretary of State website, and examining individual clinic websites. To explore possible clinic locations for non-member clinics, the investigator reviewed a web-based directory of free and reduced healthcare services (NChealthcarehelp.org), which confirmed the list of clinics provided by the NCAFC.
